# Squamous cell carcinoma transformation in mature cystic teratoma of the ovary: a systematic review

**DOI:** 10.1186/s12885-019-5393-y

**Published:** 2019-03-11

**Authors:** Congcong Li, Qing Zhang, Siying Zhang, Ruifen Dong, Chenggong Sun, Chunping Qiu, Zhiwei Zhang, Xingsheng Yang, Beihua Kong

**Affiliations:** 1 Department of Obstetrics and Gynecology, Qilu Hospital, Shandong University, 107 West Wenhua Road, Ji’nan, Shandong 250012 People’s Republic of China; 2 Gynecology Oncology Key Laboratory, Qilu Hospital, Shandong University, Ji’nan, Shandong 250012 People’s Republic of China

**Keywords:** Ovarian cancer, Mature cystic teratoma of the ovary, Squamous cell carcinoma transformation, Survival analysis

## Abstract

**Background:**

0.17–2% of mature cystic teratoma of the ovary (MCTO) undergo malignant transformation, of which 80% are squamous cell carcinoma (SCC) transformation in MCTO. We aim to investigate the clinical characteristics and treatment of SCC transformation in MCTO

**Methods:**

We systematically searched PubMed database and individual patient data about SCC transformation in MCTO were extracted. The published cases were combined with 6 cases of SCC transformation in MCTO from Qilu Hospital, Shandong University.

**Results:**

The incidence of SCC transformation in MCTO was 0.3%. A total of 435 cases of SCC transformation in MCTO were enrolled in the analysis. The mean age of diagnosis was 53.5 (range 19–87) years old. The most common clinical manifestations were abdominal pain (47.3%) and abdominal mass (26.0%). StageI,II, III and IV accounted for 50.0, 18.8, 26.8 and 4.4% of all cases, respectively. Patients with stage I had significantly better prognosis than stage II, III and IV patients (*P* < 0.01). Hysterectomy can improve overall survival (*P* < 0.01). For patients younger than 45 years old with stageIA orIC, there was no difference in mortality between fertility-sparing and radical surgery (*P* = 1.00). Adjuvant chemotherapy can improve survival in patients with advanced stage (*P* = 0.02), and chemotherapy with platinum was related to better prognosis (*P* = 0.02).

**Conclusion:**

SCC transformation in MCTO is a rare malignancy mainly occurs in older age. FIGO stage is an independent prognostic factor. Hysterectomy and platinum-based chemotherapy are associated with better survival. Fertility-sparing surgery is feasible for young patients with early stage.

**Electronic supplementary material:**

The online version of this article (10.1186/s12885-019-5393-y) contains supplementary material, which is available to authorized users.

## Background

Mature cystic teratoma of the ovary (MCTO) may occur in 10–20% of women during their lifetime [1]. The biological behavior of MCTO is benign, while 0.17–2% of MCTO may undergo malignant transformation [2]. There are various histological types of malignant transformation such as squamous cell carcinoma (SCC), adenocarcinoma, small cell carcinoma, sarcoma, malignant melanoma and mixed histology [3]. Among them SCC transformation in MCTO is most common, accounting for 80% of all malignant transformation [4].

The clinical manifestations of SCC transformation in MCTO are not specific. Tumor of early stage is often detected accidentally during physical examination or postoperative pathological examination [5], while palpable mass, bloating and abdominal pain are often present in advanced stage [6, 7]. Acute abdomen may occur due to tumor torsion or rupture [8]. Moreover, preoperative imaging investigation and laboratory tests are not specific, either.

Large-scale clinical prospective study is not feasible because of the low incidence of SCC transformation in MCTO and the published cases are scattered, and the optimal treatment for SCC transformation in MCTO remains unclear [9]. Some doctors hold the idea that since SCC originates from epithelium so its treatment should follow principles that of epithelial ovarian cancer [10, 11], some believe that the treatment should refer to SCC of other sites [12], and some suggest that since the malignancy presents on the basis of MCTO, which is a kind of ovarian germ cell tumors, the treatment can refer to that of ovarian germ cell malignancy. However, the surgical principle and postoperative adjuvant therapy of epithelial ovarian cancer, ovarian germ cell tumors and squamous cell carcinoma are different [13]. So it is of great importance to find out the most effective treatment.

In this study, we systematic review published data about SCC transformation in MCTO from 1977 to 2016 and cases from Qilu Hospital, Shandong University, aiming to further elucidate the clinical characteristics, prognostic factors and treatment of SCC transformation in MCTO and provide evidence of clinical management of this rare malignancy.

## Methods

We searched PubMed database with key words of “squamous cell carcinoma of mature cystic teratoma ovary”, “malignant transformation mature teratoma ovary”, “second tumor teratoma”, “malignant dermoid cyst ovary”, “MCTO squamous cell carcinoma”, “SCC in MCTO”, “mature cystic teratoma malignant”. Cases of SCC transformation in MCTO published from January 1977 to October 2016 were included. Only cases with specific histology, invasive behavior and available individual patient data were included (Additional file 1: Figure S1). The MCTO cases of Qilu hospital between January 2005 and October 2016 were reviewed and 6 cases SCC transformation in MCTO were included in this study. Clinicopathologic information, including age, tumor size, symptoms, stage, histological grade, surgical approach, adjuvant therapy, survival status and follow-up time were collected and analyzed. We used SPSS (version 21.0, for Windows) for statistical analysis. Data analysis was performed with descriptive statistics, chi-square test, Kaplan-Meier plots with Log-rank test, univariate and multivariate Cox proportional hazards regression model. A two-tailed *P* < 0.05 value was defined as statistically significant.

## Results

A total of 435 cases were included in this study, with 429 SCC transformation in MCTO cases from 45 case series and 54 case report [3–8, 10–12, 14–103] (Table 1), and 6 cases from Qilu Hospital, Shandong University.

**Table 1:** Summary data from published literatures on SCC transformation in MCTO

a. Case series on SCC transformation in MCTO												
Country of study	Author (year)	Number of cases	Age	Stage					Grade			
				I	II	III	IV	NA	1	2	3	NA
Japan	Yoshida et al. (2016)	2	37–64		2							2
Japan	Tazo et al. (2016)	2	45–53	2					2			
Korea	Park et al. (2015)	2	48–67		1	1						2
UK	Araujo et al.(2015)	4	35–44	2	1		1			2	2	
China	Chiang et al.(2015)	4	32–54	2		2						4
Turkey	Koc et al. (2015)	12	28–62	8	1	2	1					12
USA	Rojas et al. (2015)	2	55–71		1	1				2		
Korea	Choi et al.(2014)	4	35–51	2		2						2
Pakistan	Hannan et al. (2014)	3	50–66	2		1					3	
Thailand	Oranratanaphan et al. (2013)	4	34–70	2		2						4
UK	Powell et al. (2013)	6	42–65			4	2					6
Turkey	Ulker et al.(2012)	3	43–47	3								3
China	Chiang et al. (2011)	3	32–54	1		2				1		2
Japan	Sakuma et al.(2010)	15	29–77	8	3	3	1		5	2	3	5
India	Gupta et al. (2009)	2	30–65	2						2		
Korea	Park et al.(2008)	12	29–75	6	1	5						2
Japan	Iwasa et al.(2008)	21	32–84	14	2	4	1		6	10	5	
UK	Hurwitz et al.(2007)	12	27–69	8	2	2			1	3	8	
Japan	Yamaguchi et al.(2007)	11	29–67	7	1	2	1					11
India	Bal et al. (2007)	4	35–45					4				4
Korea	Park et al. (2007)	5	31–75	3		1	1					5
USA	Dos Santos et al. (2007)	17	37–75	8	5	4						17
Korea	Rim et al. (2006)	7	19–71	6	1				1	1	2	3
China	Wen et al. (2006)	2	32–52	2						1		1
Thailand	Tangjitgamol et al. (2003)	4	42–74	2	1	1						4
Japan	Sumi et al. (2001)	3	53–72	1	1	1						3
China	Chen et al. (2001)	3	30–65		2	1						3
Japan	Hirai et al. (2000)	3	61–72	2	1							3
Japan	Emoto et al. (2000)	5	39–73		1	5						5
China	Shen et al. (1998)	10	30–82	2	1	3	2	2				10
Japan	Yoshioka et al. (1998)	4	41–68	3		1						4
Japan	Kikkawa et al. (1998)	37	28–87	19	5	13			9	7	6	5
China	Tseng et al. (1996)	26	21–77	13	2	10	1		2	12	12	
Turkey	Zorlu et al. (1996)	3	28–42	3								3
USA	Pins et al. (1996)	16	21–75	7	6	3			2	7	7	
Japan	Hirakawa et al. (1989)	28	32–84	18	3	5	1	1	14	10	3	1
Japan	Kimura et al. (1989)	6	37–80	2	3	1			2	3	1	
Japan	Kashimura et al. (1989)	7	49–78	5	1	1						7
Netherlands	Chadha et al. (1988)	16	35–73	8	1	5	2		8	1	5	2
UK	Ribeiro et al. (1987)	6	25–64		5	1			1		2	3
Japan	Tamaya et al. (1984)	2	30–61				2					2
UK	Stamp et al. (1983)	18	36–76					18				18
Australia	Curling et al. (1979)	10	46–73	6	2	2			6	2	2	
Spain	Amerigo et al. (1978)	5	42–59	2		2	1					5
USA	Krumerman et al. (1976)	4	51–65	2	1	1						4
b. Case reports on SCC transformation in MCTO												
Country of study	Author (year)		Age		Stage			Stage				
UK	Gooneratne et al. (2015)		63		II			3				
USA	Black et al. (2015)		74		I			NA				
India	Srivastava et al. (2015)		60		II			2				
Greece	Kalampokas et al. (2014)		56		I			1				
USA	Yarmohammadi et al. (2014)		48		III			3				
India	Patni et al. (2014)		53		I			NA				
Korea	Yun et al. (2013)		30		I			1				
Turkey	Balik et al. (2013)		66		III			2				
Pakistan	Chaudhry et al. (2013)		43		I			2				
India	Mandal et al. (2012)		56		III			1				
Turkey	Avci et al. (2012)		52		I			2				
USA	Song et al. (2012)		73		II			1				
Serbia	Amidzic et al. (2012)		80		I			1				
USA	Baughn et al. (2011)		58		I			NA				
Turkey	Kahraman et al. (2011)		63		I			NA				
India	Prasad et al. (2011)		40		III			1				
Nigenia	Badmos et al. (2011)		46		II			NA				
Japan	Ito et al. (2011)		78		II			NA				
USA	Parithivel et al. (2011)		68		II			3				
USA	Alatassi et al. (2011)		49		II			NA				
India	Madan et al. (2010)		37		I			1				
Greece	Korkontzelos et al. (2010)		56		I			NA				
Japan	Hosokawa et al. (2010)		52		I			NA				
Germany	Budiman et al. (2010)		41		I			3				
Korea	Lim et al. (2009)		68		III			NA				
Iran	Shariat-Torbaghan et al. (2009)		63		IV			3				
Brazil	Silva et al. (2009)		75		I			1				
China	Wang et al. (2008)		39		III			2				
China	Ding et al. (2008)		62		III			NA				
Japan	Mekaru et al. (2008)		33		I			NA				
India	Santwani et al. (2008)		40		I			NA				
Turkey	Arioz et al. (2007)		31		II			2				
UK	Sanghera et al. (2006)		48		II			NA				
Greece	Filippakis et al. (2006)		41		I			NA				
USA	Spannuth et al. (2005)		52		II			3				
China	Lai et al. (2005)		47		III			NA				
UK	Karanjgaokar et al. (2005)		66		III			2				
UK	Mechery et al. (2004)		51		I			3				
Japan	Takemori et al. (2003)		69		II			NA				
USA	Powell et al. (2003)		67		III			NA				
Canada	Do et al. (2002)		44		II			2				
Canada	Mayer et al. (2002)		37		II			2				
USA	Noumoff et al. (2001)		36		I			3				
Japan	Takeuchi et al. (2000)		72		II			NA				
France	Kurtz et al. (1999)		34		III			NA				
Japan	Isoda et al. (1999)		56		II			NA				
USA	Lee et al. (1999)		50		II			1				
UK	As et al. (1997)		33		II			2				
USA	Griffiths et al. (1995)		76		III			2				
USA	Kung et al. (1994)		44		I			3				
USA	Rose et al. (1993)		42		III			NA				
USA	Christopherson et al. (1989)		26		III			1				
USA	Selim et al. (1984)		43		I			NA				
Japan	Mitui et al. (1983)		72		III			1				

### Retrospective chart review

From January 2005 to October 2016, there were 1836 MCTO cases in Qilu Hospital, Shandong University, 6 cases of them were SCC transformation in MCTO, accounting for 0.3% of all MCTO cases. Mean age of the 6 patients was 53.7 (range 26–68) years. Follow-up time was 3–72 months. 2 cases underwent comprehensive staging surgery of ovarian cancer. 1 patient received fertility-sparing surgery. For this patient, the diagnosis of SCC transformation in MCTO in stageIC was made after initial left ovarian tumor resection, and then she received fertility-sparing staging surgery. 3 widespread metastatic cases underwent cytoreductive surgery and 1 of them was optimally debulked (residual tumor volume less than 1 cm). 4 cases were treated with postoperative chemotherapy. All of them received TC regimen for 1–6 cycles. No case was treated with postoperative radiotherapy. Details on clinical characteristics were listed in Table 2.

**Table 2:** Clinical features of SCC transformation in MCTO from a retrospective chart review

Case	Age (years)	Symptoms	Elevated tumor marker	Diameter (cm)	Surgery	Optimal debulking	Rupture	Grade	Stage	Adjuvant therapy	Follow-up (status, months)
1	≥45	Accidental finding	–	7	TH + LSO + BPLND + omentectomy	Y	N	3	IA	TC × 1	LOST, 3
2	≥45	Pain	CA125(179.5 U/mL), CEA (6.1 ng/mL)	12	TH + BSO + omentectomy + BPLND + BPALND + peritoneal tumor resection	Y	N	2	IIB	TC × 6	NED, 72
3	≥45	Pain, distension	CA125(363.8 U/mL), CEA (25.2 ng/mL)	15	TH + BSO + appendectomy	Y	N	2	IIIB	TC × 1	DOD, 9
4	≥45	Pain, distension, fever	CA125(62.9 U/mL), SCC (7.3 ng/mL)	20	TH + BSO + omentectomy + partial peritoneal resection	N	Preoperative	2	IIIC	–	LOST, 24
5	≥45	Pain, vaginal bleeding	CA125(144.7 U/mL), SCC (15.1 ng/mL), CA199(119.1 U/mL), CEA (40.9 ng/mL)	25	TH + BSO + tumor resection + sigmoidectomy	N	Intraoperative	3	IIIC	–	DOD, 15
6	<45	Accidental finding	CA125 (42.8 U/mL), CA199(97.1 U/mL)	5	Initial surgery: Left ovarian cystectomy Restaging surgery: LSO + omentectomy + BPLND + BPALND	Y	N	3	IC	TC × 3	NED, 8

*TH* total hysterectomy, *LSO* left salpingo-oophorectomy, *BSO* bilateral salpingo-oophorectomy, *BPLND* bilateral pelvic lymph node dissection, *BPALND* bilateral para-aortic lymph node dissection, *TC* taxol/carboplatin, *Y* yes, *N* no, *DOD* die of disease, *NED* no evidence of disease

**Fig. 1:**
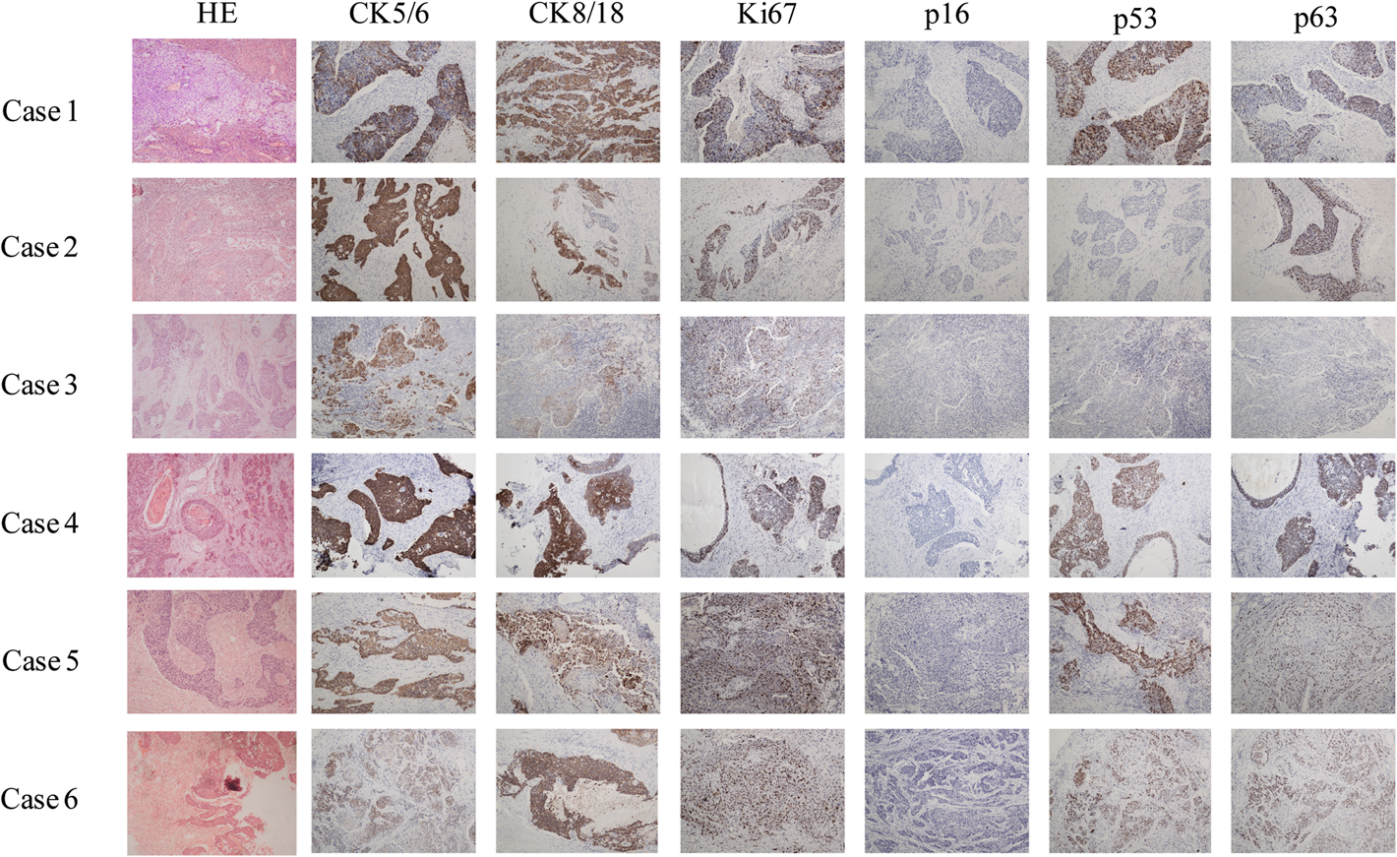
Histological features of SCC transformation in MCTO from a retrospective chart review

Imaging investigation indicated cystic-solid mass with blood flow signal and enhancement. Grossly, the tumor consisted of cystic and solid components. There were typical components of MCTO such as hair, oil, sebaceous glands and epidermis. Microscopically, there were increased layers of squamous epithelium, disorderly arranged cells, squamous cells with atypia, irregular nuclei and necrosis (Fig. 1). All 6 cases were positive staining for p63, negative staining for p16, moderate to strong positive staining for Ki67 and alteration in p53. Detailed immunohistochemical panel is listed in Table 3 and Fig. 1.

**Table 3:** Immunohistochemical panel of SCC transformation in MCTO from a retrospective chart review

Antibody	Source	Dilution	Case 1	Case 2	Case 3	Case 4	Case 5	Case 6
Ki67	Abcam	1:250	++	++	++	+++	++	++
p16	Abcam	1:100	–	–	–	–	–	–
p63	Abcam	1:500	+	++	++	++	+	++
CK8/18	Dako	prediluted	+++	+++, 5%	++	+++	+++	+++
CK5/6	Dako	prediluted	+++	++	+++	+++	++	+++
p53	Abcam	1:100	+, focal	+++	–	++	++	+++

The antibody reaction was graded according to the intensity od staining: - (negative), + (weak), ++(moderate), +++ (strong)

### Systematic review

**Fig. 2:**
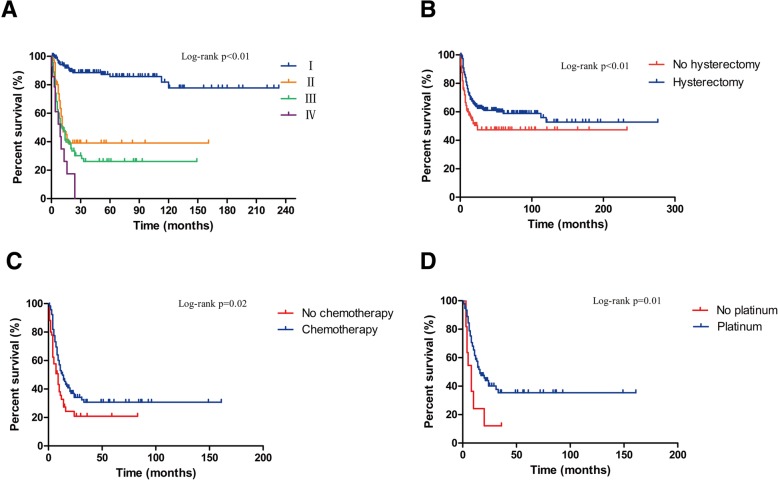
**a**. FIGO stage and overall survival. **b**. hysterectomy and overall survival. **c**. chemotherapy and overall survival for stage II, III, IV. **d**. chemotherapy with platinum and overall survival for stage II, III, IV

Of the 435 cases of SCC transformation in MCTO, follow-up information was available for 363 cases and the follow-up time ranged from 0.4 months to 276 months. 146 (40.2%) cases ended up with death during the follow-up. Mean age of diagnosis was 53.5 (range 19–87) years. 121(27.9%) were<45 year old while 313(72.1%) were ≥ 45 years old (Table 4). Age ≥ 45 years was related with worse prognosis compared with patients<45 (*P* < 0.01) (Table 5). Mean tumor size was 14.8(range 3.5–40.0) cm. Overall survival of tumor ≤10 cm and>10 cm were of no difference (Table 5). Median (Q25, Q75) preoperative SCC-Ag was 7.4 (3.0, 20.0) ng/mL, CA125 was 64.4 (34.2, 143.0) U/mL, CA19–9 was 144.0 (45.1, 943.5) U/mL and CEA was 6.9 (2.5, 23.0) ng/mL. Abdominal pain and palpable abdominal mass were two major clinical manifestations of SCC transformation in MCTO, occurring in 47.3 and 26% of all cases, respectively. StageI,II, III and IV accounted for 50.0, 18.8, 26.8 and 4.4% of all cases, respectively (Table 4). Compared with stage I, stage II, III and IV were associated with worse prognosis (*P* < 0.01) (Table 5, Fig. 2a). 5-year overall survival was 85.8, 39.1, 26.2 and 0% for stage I, II, III, and IV, respectively. There was no interaction between histology grade and survival (Table 5).

**Table 4:** Clinical features of SCC transformation in MCTO

	Results
Age, years (*n* = 434)^a^	53.5 (13.9, 19–87)
<45 years	121
≥45 years	313
Tumor size, cm (*n* = 316)^a^	14.8 (5.9, 3.5–40)
Preoperative tumor markers^b^	
SCC-Ag, ng/mL (*n* = 78)	7.4 (3.0, 20.0)
CA125, U/mL(*n* = 104)	64.4 (34.2, 143.0)
CA19–9,U/mL (*n* = 65)	144.0 (45.1, 943.5)
CEA, ng/mL (*n* = 51)	6.9 (2.5, 23.0)
Clinical manifestation (*n* = 204)	
Abdominal/pelvic pain	139
Mass	53
Abdominal bloating	49
Physical examination	10
Urinary frequency	6
Weight loss	11
Change in bowel habits	20
Fever	7
FIGO staging (*n* = 414)	
I	207
II	78
III	111
IV	18
Histological grade (*n* = 203)	
1	54
2	84
3	65

^a^Mean (SD, range); ^b^ Median (Q25, Q75)

**Table 5:** Clinical features and overall survival of SCC transformation in MCTO

Variable	Univariate HR (95% CI)	*P* value
Age (*n* = 370)		
<45 (*n* = 105)	Reference	
≥45 (*n* = 265)	1.91 (1.26–2.89)	<0.01^*^
Diameter (cm) (*n* = 265) (*n* = 265)		
≤10 (*n* = 67)	Reference	
>10 (*n* = 198)	1.08 (0.70–1.68)	0.73
Stage (*n* = 348)		
I (*n* = 172)	Reference	
II (*n* = 70)	6.92 (3.98–12.06)	<0.01^*^
III (*n* = 92)	9.32 (5.58–15.59)	<0.01^*^
IV (*n* = 14)	16.45(7.95–34.02)	<0.01^*^
Grade (*n* = 169)		
1 (n = 43)	Reference	
2 (*n* = 69)	1.60 (0.81–3.15)	0.17
3 (*n* = 57)	1.76 (0.89–3.49)	0.10

Cox proportional hazard regression model analysis

As for treatment, hysterectomy can reduce death risk (*P* < 0.01) (Table 6, Fig. 2b) whereas lymphadenectomy did not improve survival. Omentectomy did not improve survival with univariant analysis, while after adjusted with International Federation of Gynecology and Obstetrics (FIGO) stage, omentectomy was associated with better prognosis (*P* = 0.04) (Table 6). 24 of the 51 patients younger than 45 years old with stageIA orIC underwent fertility-sparing surgery. There was no difference in mortality between fertility-sparing and radical surgery (*P* = 1.00).

**Table 6:** Surgery modality and overall survival of SCC transformation in MCTO

Surgery	Univariate HR (95% CI)	*P* value	Stage-adjusted HR (95% CI)	*P* value
Hysterectomy (325)				
Yes (232) vs No (93)	0.60 (0.42–0.85)	< 0.01^*^	0.51 (0.36–0.74)	< 0.01^*^
\Lymphadenectomy (325)				
Yes (69) vs No (256)	0.69 (0.43–1.09)	0.11	0.76 (0.47–1.22)	0.25
Omentectomy (313)				
Yes (120) vs No (193)	1.04 (0.72–1.48)	0.85	0.66 (0.45–0.98)	0.04

Cox proportional hazard regression model analysis

Efficacy of postoperative adjuvant therapy was analyzed in a subgroup of SCC transformation in MCTO with stage II, III and IV. Adjuvant chemotherapy can improve survival in patients with advanced stage (*P* = 0.02) (Table 7, Fig. 2c), and chemotherapy with platinum was related to better prognosis compared with other drugs (*P* = 0.02) (Table 8, Fig. 2d). However, radiotherapy and chemoradiotherapy did not improve survival (Table 7).

**Table 7:** Treatments and overall survival of SCC transformation in MCTO of stage II, III and IV

Treatment	Univariate HR (95% CI)	*P* value	Stage-adjusted HR (95% CI)	*P* value
Chemotherapy (170)				
Yes (118) vs No (52)	0.60 (0.40–0.91)	0.02^*^	0.56 (0.37–0.86)	0.01^*^
Radiotherapy (170)				
Yes (50) vs No (120)	0.80 (0.52–1.21)	0.29	0.89 (0.57–1.38)	0.60
Chemoradiotherapy (170)				
Yes (31) vs No (139)	0.71(0.43–1.16)	0.17	0.73 (0.44–1.21)	0.22

Cox proportional hazard regression model analysis

**Table 8:** Chemotherapy regimen and overall survival of SCC transformation in MCTO of stage II, III and IV

Drug (*n* = 103)	Univariate HR (95% CI)	*P* value	Stage-adjusted HR (95% CI)	*P* value
Platinum derivatives (n = 92)	0.43 (0.21–0.87)	0.02^*^	0.41 (0.20–0.84)	0.02^*^
Taxanes (*n* = 29)	1.09 (0.61–1.98)	0.78	1.10 (0.60–2.00)	0.76
Vinca alkaloids (*n* = 21)	0.59 (0.30–1.20)	0.14	0.48 (0.23–1.01)	0.05
Alkylating agents (*n* = 15)	1.05 (0.53–2.09)	0.88	0.84 (0.38–1.84)	0.66
5-FU (n = 16)	0.77 (0.36–1.62)	0.50	0.82 (0.39–1.75)	0.61
Bleomycin (*n* = 36)	0.94 (0.55–1.61)	0.82	0.85 (0.48–1.50)	0.57
VP-16 (*n* = 12)	0.76 (0.33–1.78)	0.53	0.82 (0.35–1.92)	0.64

Cox proportional hazard regression model analysis

## Discussion

SCC transformation in MCTO is the most common malignant transformation of MCTO [4] and is also the most common cause of SCC of the ovary [9, 104]. SCC transformation in MCTO may be a continuous process of squamous metaplasia, atypical hyperplasia, carcinoma in situ, interstitial infiltration and invasive carcinoma [14, 105]. The mean age of MCTO patients without malignant transformation is 32.7 years [1] while the mean age of SCC transformation in MCTO in our study is 53.5 years. Some cases have a history of MCTO [19, 70], suggesting that unmanaged MCTO may undergo malignant transformation. SCC transformation in MCTO may be associated with high-risk human papilloma virus (HPV) infection [15], and alterations in p53 and p16 may be involved in the process of malignant transformation [16, 17, 106]. A recent next-generation sequencing analysis indicated that TP53 mutation was detected in 80% of SCC transformation in MCTO cases and mutation in TP53 correlated with better prognosis. PIK3CA and CDKN2A were altered in 52 and 44% cases, respectively [107].

In this study, 50% of cases are diagnosed in FIGO stage I. The proportion of stage I is much higher than that of all ovarian cancer, which is 15% [108]. More SCC transformation in MCTO cases can be detected in early stage because the malignant component originates from epithelium of preexistent MCTO [14], which is a gradual process, and is often found unexpectedly after resection of adnexal mass [1]. The same as epithelial ovarian cancer, FIGO stage is an independent prognostic factor for SCC transformation in MCTO. In this study, the 5-year survival rate of stage I patients is 85%, while in patients with stage II, III, the 5-year survival rate is less than 50%. So early detection is of great value and can improve the prognosis of SCC transformation in MCTO.

Comprehensive staging surgery is the standard treatment for ovarian cancer. Hysterectomy and omentectomy appear to improve survival of SCC transformation in MCTO, while lymph node dissection does not affect overall survival in our analysis. This observation suggests that local dissemination is an important metastasis manner of SCC transformation in MCTO. The cases included in this study are retrospective and are from multiple medical centers. What’s more, some cases do not point out the exact scope of lymph node dissection. Since lymph node dissection is part of comprehensive staging surgery, we are in favor of performing lymphadenectomy despite the negative result in this study.

For patients with epithelial ovarian cancer in stages IA/IC, unilateral salpingo-oophorectomy is an optional treatment. For patients with malignant ovarian germ cell tumors, regardless of stage, fertility-sparing surgery can be done and after delivery a comprehensive operation should be conducted [13]. Our study suggests that for SCC transformation in MCTO, it is safe and feasible to perform fertility-sparing surgery in patients younger than 45 years of age old with stage IA/IC. There are cases of successful pregnancies after fertility-sparing surgery [57, 74, 83].

The recommend initial chemotherapy regimen for ovarian germ cell tumor and for epithelial ovarian cancer is bleomycin/etoposide/cisplatin (BEP) and paclitaxel/carboplatin (TC), respectively. Currently there is no recognized first-line adjuvant therapy for SCC transformation in MCTO, though chemotherapy can improve prognosis of patients with SCC transformation in MCTO of advanced stage. Hackethal et al. suggest that chemotherapy with alkylating agents is related to better prognosis in patients with SCC transformation in MCTO [109]. However, in our study with the analysis of patients of stage II, III and IV, alkylating agents did not promote overall survival compared with others. We recommend individualized and integrated treatment based on platinum-based chemotherapy.

The histology of SCC transformation in MCTO is squamous cell carcinoma and genetic study indicates that SCC transformation in MCTO has features in common with other SCC [107], which are often sensitive to radiotherapy. However, our results suggested that radiotherapy does not improve prognosis of SCC transformation in MCTO. Whenever applied, doctors should pay attention to complications of radiotherapy [110].

This study has some limitations. Firstly, since the analysis is mostly based on review on published data, we cannot avoid the publication bias. Secondly, although preoperative detection of SCC transformation in MCTO is of great value, in our study we do not conduct the analysis because only a few papers reported the imaging features and serum markers [111, 112]. The size of invasive component, which is possibly related to prognosis, is not available due to the retrospective study design. Moreover, few publications report cycles of chemotherapy, thus we do not investigate the optimal course of treatment. We recommend presentation of such information whenever reporting cases. Lastly, our results should be treated with caution, as the included studies span a prolonged time period (~ 40 years) during which changes in medical and therapeutic strategies may have changed.

## Conclusions

SCC transformation in MCTO is a rare malignancy mainly occurs in older age. Early detection is important for better prognosis. Hysterectomy and platinum-based chemotherapy are associated with better survival. Fertility-sparing surgery is feasible for young patients with early stage. Due to the retrospective study design and limited data available, the results should be interpreted with caution. More reports are in need to elucidate the biology of SCC transformation in MCTO.

## Additional file

Additional file 1: **Figure S1.** The flow chart of study selection and case inclusion. **Table S1.** Stage of different treatment group in survival analysis. (DOCX 40 kb)

## Abbreviations

BEP: Bleomycin/etoposide/cisplatin
BPALND: Bilateral para-aortic lymph node dissection
BPLND: Bilateral pelvic lymph node dissection
BSO: Bilateral salpingo-oophorectomy
DOD: Die of disease
FIGO: International Federation of Gynecology and Obstetrics
HPV: Human papilloma virus
HR: Hazard ratio
LSO: Left salpingo-oophorectomy
MCTO: Mature cystic teratoma of the ovary
NED: No evidence of disease
SCC: Squamous cell carcinoma
TC: Paclitaxel/carboplatin
TH: Total hysterectomy
