# Supplementary material for: Squamous cell carcinoma transformation in mature cystic teratoma of the ovary: a systematic review
Source: BMC Cancer. 2019 Mar 11;19:217. doi: 10.1186/s12885-019-5393-y (PMC6417039; doi:10.1186/s12885-019-5393-y)
Supplement: Supplementary file 1 — Figure S1. The flow chart of study selection and case inclusion. Table S1. Stage of different treatment group in survival analysis. (DOCX 40 kb) [file 12885_2019_5393_MOESM1_ESM.docx]

**Search strategy (PubMed):**

Search (((((((((malignant transformation mature teratoma ovary) OR squamous cell carcinoma arising from mature cystic teratoma ovary) OR second tumor teratoma)) OR malignant dermoid cyst of the ovary) OR MCT squamous cell carcinoma) OR SCC in MCT) OR mature cystic teratoma malignant) AND ( "1977/01/01"[PDat] : "2016/10/31"[PDat] )) Sort by: Relevance

**Figure S1**

435 cases from 45 case series, 54 case reports, and our institution were included in this study

Cases from our institution (n=6)

Duplicate cases (n=2)

No sufficient individual data (n=7)

Review (n=2)

Autopsy (n=1)

Molecular research (n=1)

Reference from reviews (n=6)

Full text screening (n=106)

Different pathological types (n=20)

Mixed pathology (n=5)

Not in English (n=12)

Review (n=3)

Carcinoma in situ (n=3)

Not relevant (n=10)

Full text not available (n=16)

Abstract screening (n=175)

Title screening (n=1843)

Records identified through PubMed search (n=1843)

Table S1. Stage of different treatment group in survival analysis

| **Treatment** | **Yes** | | | |  | **No** | | | | ***P*** |
| --- | --- | --- | --- | --- | --- | --- | --- | --- | --- | --- |
|  | **I** | **II** | **III** | **IV** |  | **I** | **II** | **III** | **IV** |  |
| **Hysterectomy (325)** | 117 | 48 | 59 | 8 |  | 43 | 16 | 28 | 6 | 0.44 ^1^ |
| **Lymphadenectomy (325)** | 35 | 14 | 19 | 1 |  | 125 | 50 | 68 | 13 | 0.70 ^1^ |
| **Omentectomy (313)** | 46 | 24 | 45 | 5 |  | 114 | 33 | 37 | 9 | <0.01 ^2^ |
| **Chemotherapy (170)** |  | 45 | 66 | 7 |  |  | 24 | 22 | 6 | 0.20 ^1^ |
| **Radiotherapy (170)** |  | 27 | 21 | 2 |  |  | 42 | 67 | 11 | 0.07 ^1^ |
| **Chemoradiotherapy (170)** |  | 13 | 16 | 2 |  |  | 56 | 72 | 11 | 1.00 ^1^ |

^1^ Fisher’s exact test; ^2^ Pearson chi-square test
